# NgAgo-based *fabp11a* gene knockdown causes eye developmental defects in zebrafish

**DOI:** 10.1038/cr.2016.134

**Published:** 2016-11-11

**Authors:** Jialing Qi, Zhangji Dong, Yunwei Shi, Xin Wang, Yinyin Qin, Yongming Wang, Dong Liu

**Affiliations:** 1 Co-innovation Center of Neuroregeneration, Jiangsu Key Laboratory of Neuroregeneration, Nantong University, Nantong, Jiangsu 226001, China; 2 The State Key Laboratory of Genetic Engineering and MOE Key Laboratory of Contemporary Anthropology, School of Life Sciences, Fudan University, Shanghai 200433, China

## Dear Editor,

A recent report of genome editing using *Natronobacterium gregoryi* Argonaute (NgAgo) with guide DNA (gDNA) in human cells^1^ prompted us to explore the utility of this protein for *in vivo* genetic manipulation in zebrafish *(Danio rerio)*. Zebrafish is a model organism that offers several distinct advantages for studying genetics, developmental biology, vascular biology and disease modeling. In the last several years, loss-of-function genomic editing techniques, including zinc-finger nucleases (ZFNs)^2,3^, artificial transcription activator-like effector nucleases (TALENs)^4,5^ and clustered regularly interspaced short palindromic repeats (CRISPR)/CRISPR-associated (Cas) 9 system (CRISPR/Cas9)^6,7^, have been adopted for zebrafish. Recently the gDNA/NgAgo system has elicited much interest because of some unique advantages: low tolerance to guide-target mismatch, minimum off-target effects, and easy to design^1^. Here we investigated whether the gDNA/NgAgo system could be used to manipulate zebrafish genes *in vivo* using *fabp11a* as a test case.

**Figure 1:**
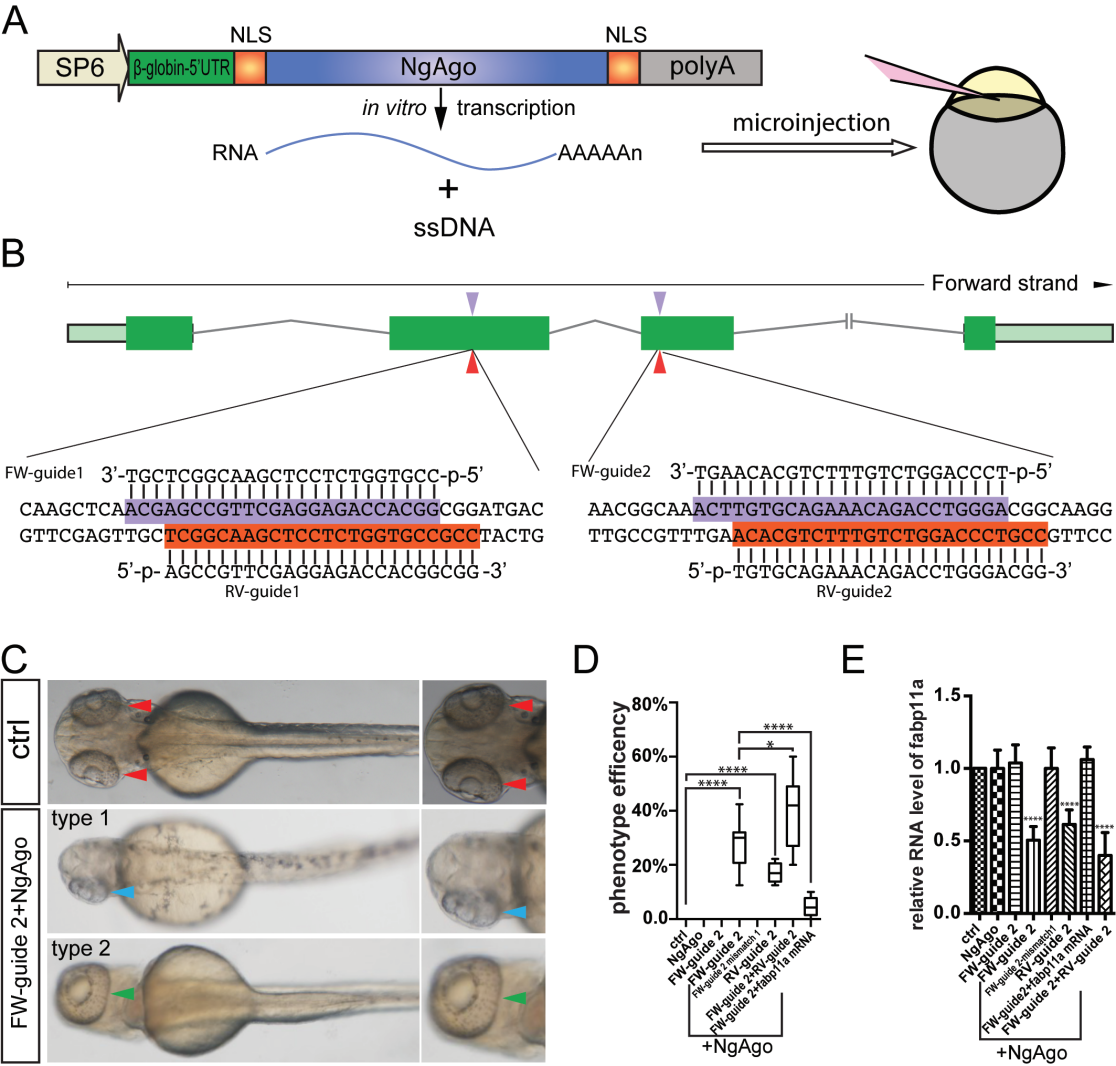
NgAgo-based *fabp11a* gene knockdown causes eye developmental defects in zebrafish. **(A)** Schematic diagram showing the structure of *NgAgo-2nls* for *in vitro* transcription and microinjection. **(B)** Schematic diagram showing guide DNA targeting sites (indicated by arrowhead) in the exons of *fabp11a* gene. The targeting sequences are highlighted in purple and red. **(C)** Microscopy analysis of eyes in control and FW-guide DNA 2 and *NgAgo-2nls* mRNA coinjected embryos, dorsal view, with the head regions enlarged and shown on the right. Red arrowheads indicate normal paired eyes. Blue arrowhead indicates one single eye in FW-guide 2 and *NgAgo-2nls* mRNA coinjected embryo. Green arrowhead indicates a single fused large eye. **(D)** Statistical analysis of phenotype efficiency in control (0%); *NgAgo-2nls* mRNA only (0%); FW-guide 2 only (0%); FW-guide 2 and *NgAgo-2nls* mRNA (29.6%); FW-guide 2 mismatch 1 and *NgAgo-2nls* mRNA (0%); RV-guide 2 and *NgAgo-2nls* mRNA (15.4%); FW-guide 2, RV-guide 2 and *NgAgo-2nls* mRNA (39.7%); FW-guide 2, *NgAgo-2nls* mRNA and *fabp11a* mRNA injected embryos (4.6%). One-Way ANOVA; ^****^ *P* < 0.0001; ^*^ *P*< 0.05. **(E)** Relative mRNA levels of zebrafish *fabp11a* in 30 hpf control; *NgAgo-2nls* mRNA only; FW-guide 2 only; FW-guide 2 and *NgAgo-2nls* mRNA; FW-guide 2 mismatch 1 and *NgAgo-2nls* mRNA; RV-guide 2 and *NgAgo-2nls* mRNA; FW-guide 2, *NgAgo-2nls* mRNA and *fabp11a* mRNA; FW-guide 2, RV-guide 2 and *NgAgo-2nls* mRNA injected embryos. One-Way ANOVA; ^****^ *P* < 0.0001.

Fatty acid binding protein 11a (Fabp11a) is a member of the family of intracellular FABPs, which play important roles in regulating glucose and lipid homeostasis as well as inflammation^8,9^. So far, the role of Fabp11a in zebrafish embryonic development remains unclear. To investigate whether 5′-phosphorylated single-strand (ss) DNA can direct NgAgo to disrupt endogenous genes *in vivo* in zebrafish, we constructed a codon-optimized NgAgo for zebrafish with nuclear localization signal (NLS) peptides at both N-terminus and C-terminus (*NgAgo-2nls*, Figure 1A and Supplementary information, Data S1). Two 24 bp 5′-phosphorylated ssDNA oligos, forward guide-1 (FW-guide 1) and FW-guide 2 (Supplementary information, Table S1A, which contains other gDNAs used in this study), targeting the second exon and third exon of *fabp11a* gene (Figure 1B) were synthesized; and each was coinjected with *NgAgo-2nls* mRNA into 1-cell stage embryos (Figure 1A). Interestingly, we observed that approximately 30% of the injected embryos displayed severe eye phenotypes, either having one very small eye and one relative normal size eye (type 1), or having one large fused eye on the top of the head like Cyclops (type 2), at 30 hours post fertilization (hpf) (Figure 1C and 1D, Supplementary information, Figure S1A(A-C)).

To investigate whether the eye phenotype was caused by genetic mutations in *fabp11a* gene, we extracted genomic DNA from embryos with abnormal eye phenotype and performed polymerase chain reaction (PCR) amplification of the targeted region. Unexpectedly, Sanger sequencing did not uncover any DNA sequence alteration in the targeted region from 57 embryos with eye phenotype we analyzed (data not shown). Instead of DNA mutations, real time reverse transcriptase (RT)-PCR analysis revealed that the *fabp11a* gene expression was down-regulated (Figure 1E and Supplementary information, Figure S1A(D)). In contrast, we did not find any specific phenotype in only *NgAgo-2nls* mRNA- or gDNA-injected embryos (Figure 1D), suggesting that the phenotype was caused by gDNA/NgAgo complex. Coinjection of *NgAgo-2nls* mRNA with two individual mismatched oligos (Supplementary information, Table S1) did not cause the eye developmental defect (Figure 1D and data not shown), suggesting specificity of the gDNA/NgAgo system. We further designed two reversed gDNAs: RV-guide 1 and 2 complementary to FW-guide 1 and FW-guide 2, respectively. Coinjection of RV-guide 1 or 2 with *NgAgo-2nls* mRNA into 1-cell stage embryos resulted in abnormal eye development in approximately 15% of embryos (Figure 1D and data not shown). Real time RT-PCR results revealed the reduction of *fabp11a* expression (Figure 1E). The abnormal eye phenotype caused by gDNA/NgAgo system could be rescued by coinjection of *fabp11a* mRNA (Figure 1D).

To confirm that gDNA/NgAgo system could knock down genes as a general principle in zebrafish, we tested another gene *ta* (*no tail*/*ntl* or *brachyury*). *ta* encodes an essential T-box transcription factor required for proper mesoderm formation; zebrafish embryos homozygous for a null *ta* mutation lack the notochord and tail^10,11^. Two 24 bp gDNAs were individually injected with *NgAgo-2nls* mRNA into 1-cell stage embryos; and, at 30 hpf, around 30% injected embryos showed typical *no tail* phenotype, whereas coinjection of mismatched gDNAs with *NgAgo-2nls* mRNA resulted in normal embryos (Supplementary information, Figure S1B and data not shown). 40 of the *no tail*-like embryos were analyzed for insertion/deletion (indel). We did not detect any mutation in the target region by Sanger sequencing (data not shown). Real time RT-PCR revealed that *ta* expression was down-regulated (Supplementary information, Figure S1B(D)). Furthermore we tested three more genes, *kdrl*, *lama1* and *flt1*. The gDNA/NgAgo system significantly reduced the expression of these genes and caused specific phenotypes similar to previously reported^12,13,14^ (Supplementary information, Figure S1C-S1E).

To test whether the length of guide DNA can influence knockdown efficiency, we used gDNAs of various lengths (20-27 nt) to target *fabp11a* and *ta*. We found that the shorter gDNAs displayed higher knockdown efficiency for both genes (Supplementary information, Figure S1F).

We also investigated whether NgAgo could generate indels under certain conditions. We injected NgAgo mRNA mixed with two complementary gDNAs targeting *fabp11a* and found that the injection caused abnormal eye phenotype but failed to introduce indels (Figure 1D and 1E). Recent report showed that gDNA/NgAgo could generate 11.2%-41.3% indels in human cells at 37 °C^1^, whereas in our experiments described above the zebrafish were maintained at 28.5 °C. Temperature might be a reason that influences nuclease activity of NgAgo. It has been reported that incubation at a temperature above 32.5 °C may cause malformations of the zebrafish embryo^15^. We found more than half of the embryos developed normally when they were incubated at 37 °C continuously until phenotypic examination. Under this condition, gDNA/NgAgo injection still failed to introduce indels in *fabp11a* in 38 embryos exhibiting eye phenotype and in *ta* in 50 embryos exhibiting *no tail*-like phenotype (data not shown). Additionally, we mutagenized two aspartic acid residues in NgAgo obtaining NgAgo-D663A, NgAgo-D738A and NgAgo-D663A-D738A, which are predicted to lack the catalytic activity of NgAgo. Coinjection of each mutant *NgAgo* mRNA with *fabp11a* FW-gDNA2 efficiently caused the eye developmental defect, with phenotype efficiency of 28.7%, 29.5% and 23.9%, respectively (Supplementary information, Figure S1G(A)). Coinjection of the mutants with *ta* FW-gDNA1 caused *no tail*-like phenotype with efficiency of 32.5%, 33.8% and 29.8%, respectively (Supplementary information, Figure S1G(B)). We could not detect any indel either in *fabp11a* in 25 *NgAgo-D663A-D738A* mRNA- and *fabp11a* FW-gDNA 2-injected embryos exhibiting eye phenotype, or in *ta* in 25 *NgAgo-D663A-D738A* mRNA- and *ta* FW-gDNA 1-injected embryos exhibiting *no tail*-like phenotype.

To confirm that the eye developmental defect was caused by *fabp11a* knockdown, we carried out further investigation. First, we examined the expression pattern of *fabp11a* at early developmental stages of zebrafish embryos. Whole mount *in situ* hybridization (WISH) analysis demonstrated that *fabp11a* is specifically and highly expressed in the eye field (optic primordium) (Supplementary information, Figure S1H), suggesting *fabp11a* plays important roles in eye development from an early stage. We next knocked down *fabp11a* using a translation-blocking morpholino (Supplementary information, Table S1A), whose efficiency was validated (data not shown); and *fabp11a* morphants showed similar eye defects (Supplementary information, Figure S1I). Interestingly, the knockdown efficiency of gDNA/NgAgo system is higher than that of the morpholino-mediated approach. In addition, we knocked out *fabp11a* using CRISPR/Cas9 system. We used two gRNAs to target *fabp11a* locus, and coinjected *cas9* mRNA with individual gRNA into one-cell embryos. Both gRNA1 and gRNA2 efficiently induced indels in *fabp11a* locus with efficiency of 60% and 42.9%, respectively (Supplementary information, Figure S1J(A-D)). gRNA1 resulted in severe eye developmental defect in approximately 30% embryos (Supplementary information, Figure S1J(E and F)); about 10% of total embryos showed extreme eye phenotype with both eyes missing at 48 hpf. In addition, the established *fabp11a* mutant embryos showed very similar eye developmental defect (Supplementary information, Figure S1J(G)) in the F1 generation, suggesting the *fabp11a* mutations and the eye abnormal phenotype are heritable.

In summary, our study shows that gene knockdown is the main mechanism by which gDNA/NgAgo affects gene function in zebrafish. This is supported by results of the experiments using presumably catalytically dead NgAgo. In addition, we have failed to detect any mutation in all the embryos we examined. Since the catalytically inactive (dead) Cas9 (dCas9):sgRNA complex could efficiently inhibit gene expression through binding to the coding sequence^16^, we hypothesize that gDNA/NgAgo may bind to a target gene to block its transcription. Overall, we suggest that the gDNA/NgAgo system provides an alternative strategy for gene knockdown in zebrafish.

## Supplementary Information

Supplementary information, Data S1 Materials and Methods

Supplementary information, Table S1 The sequences were used for *fabp11a*

Supplementary information, Figure S1 NgAgo based *fabp11a* gene knockdown caused eye developmental defects in zebrafish.
