# Supplementary material for: NgAgo-based fabp11a gene knockdown causes eye developmental defects in zebrafish
Source: Cell Res. 2016 Nov 11;26(12):1349–52. doi: 10.1038/cr.2016.134 (PMC5143420; doi:10.1038/cr.2016.134)
Supplement: Supplementary information, Data S1 — Materials and Methods [file cr2016134x1.pdf]

Supplementary information, Data S1

Materials and Methods

Zebrafish embryos

Embryos were obtained through natural mating (AB line) and maintained at 28.5 °C. Embryonic stages were defined as described in our previous work [1, 2]. Zebrafish embryos for *in situ* hybridization were collected at various stages, fixed with 4% paraformaldehyde (PFA) in phosphate-buffered saline (PBS) over night at 4 °C or 2 h at room temperature, washed with PBST, dehydrated in ethanol and stored at -20 °C until use. Embryos at stages earlier than 24 hpf were dechorionated after fixation, prior to storage. For 37 °C treatment, the fertilized eggs produced under standard condition were incubated in 37 °C incubator. The *Tg(kdrl:EGFP)* and *Tg(kdrl:mCherry)* lines were used as previously described [1, 3].

RNA extraction, reverse transcription, and quantitative PCR

Embryos and tissues were homogenized and frozen in TRIzol Reagent (Invitrogen) and stored at -80 °C. The RNA was extracted following the manufacturer's instruction. 1 μg of RNA was reverse transcribed into cDNA by the use of Transcriptor First Strand cDNA Synthesis Kit (Roche) according to the manufacturer's instructions. Synthesized cDNA was stored at -20 °C. All PCR amplifications were carried out in a total volume of 20 or 50 μl using specific primers and Advantage 2 Polymerase Kit (Clontech). The primers used for PCR, RT-PCR and real time PCR are listed in Supplementary information, Table S1B. Quantitative PCR was carried out in triplicate

using the FastStart Universal SYBR Green Master Mix (Roche Applied Science) on a real-time PCR detection system (StepOne™ Real-Time PCR Systems).

Whole mount *in situ* hybridization (WISH) and microscopy imaging

The 505 bp coding sequence for zebrafish *fabp11a* (Ensembl Transcript ID: ENSDART00000021798) was amplified and subcloned into pGEM®-T Easy Vector (Promega Corporation). The primers used for PCR, RT-PCR and real time PCR are listed in Supplementary information, Table S1B. Digoxigenin (DIG)-labeled RNA sense and antisense probes were made from the linearized plasmids according to the manufacturer's protocol using the DIG RNA Labeling Kit (SP6/T7) (Roche). The *in situ* hybridization procedure was the same as described in our previous work [2]. Pictures were taken with an Olympus DP70 camera on an Olympus stereomicroscope MVX10.

NgAgo mutation and bioinformatical analysis

Catalytic sites in NgAgo were predicted by protein sequence alignment of NgAgo, PfAgo (protein sequence retrieved from http://www.ebi.ac.uk/pdbe/entry/pdb/1Z25), TtAgo (http://www.ebi.ac.uk/pdbe/entry/pdb/4N47) and human Ago2 (http://www.ebi.ac.uk/pdbe/entry/pdb/4W5N). The Vector NTI was used for multiple Ago protein sequence alignment (http://www.thermofisher.com/cn/zh/home/life-science/cloning/vector-nti-software.ht

ml). PfAgo was reported to contain two D residues (D558, D628) that are essential for the DNA digestion [4]. By alignment of NgAgo, PfAgo, TtAgo and human Ago2, we found that these two D residues are conserved in Ago proteins. Predicted catalytic sites (D663 and D738) were mutated to Alanine by overlapping PCR. Briefly, the left and the right fragments containing designed point mutation were prepared by PCR using pCS2+-NLS-Ago as the template (primers SP6 and NgAgoD663mutR for the D663A-left-fragment, NgAgoD663F and M13R for the D663A-right-fragment; SP6 and NgAgoD738mutR for the D738A-left-fragment, NgAgoD738F and M13R for the D738A-right-fragment). Then corresponding left and right fragments were mixed to make the overlapping templates (D663A-left+D663A-right, D738A-left+D738A-right), which were amplified using SP6 and M13R, each to produce a 3135bp overlapping PCR product (NgAgo-D663A and NgAgo-D738A, respectively) ready for *in vitro* transcription. Double mutant NgAgo-D663A-D738A was prepared using the D738A primers (specificly SP6 and NgAgoD738R for the D663A-D738A-left-fragment, NgAgoD738F and M13R for the D738A-right-fragment, and then SP6 and M13R for the overlapping reaction with mixed D663A-D738A-left-fragment and D738A-right-fragment as the overlapping template) as described above but using the freshly prepared NgAgo-D663A amplicon as template instead of pCS2+-NLS-Ago in the left- and right- reactions. NgAgo-D663A, NgAgo-D738A and NgAgo-D663A-D738A were then purified using AxyPrep PCR Clean-up Kit (Axygen) and transcribed into mRNA using mMESSAGE mMACHINE Transcription Kit (Ambion). The mutation sequences

were validated by Sanger sequencing.

Primer name	Sequence
SP6	5’-ATTTAGGTGACACTATAG-3’
NgAgoD663R	5’-agatctgctcacgGcgattccgatgaacatatctg-3’
NgAgoD663F	5’-ttcatcggaatcgCcgtgagcagatcttatccaga-3’
NgAgoD738R	5’-gttcatgaatccgGctctgtggatcacgatgtgtg-3’
NgAgoD738F	5’-gtgatccacagagCcggattcatgaacgaggacct-3’
M13R	5’-CAGGAAACAGCTATGACC-3’

Injection of mRNAs, morpholino and DNA oligos

Morpholino antisense oligomers (MO, Gene Tools) were prepared according to the manufacturer’s protocol. All the 5’-p-DNA oligos were purchased from Thermo Fisher Scientific and dissolved in Ultrapure water (Thermo Fisher Scientific). *NgAgo*, *Cas9* and *fabp11* mRNAs were synthesized *in vitro* using the linearized constructs as templates with SP6/T7 mMESSAGE mMACHINE Kit (Ambion), purified with RNeasy Mini Kit (Qiagen), and dissolved in RNase free Ultrapure water (Thermo Fisher Scientific). The sgRNAs were synthesized by the MAXIscript T7 Kit (Ambion), and were purified with RNeasy Mini Kit (Qiagen), and dissolved in RNase free Ultrapure water (Thermo Fisher Scientific). The microinjection dosages are listed in the following: *fabp11a* MO 0.01 pM/embryo; *flt1* MO 0.001 pM/embryo; control MO 5 ng/embryo; *NgAgo* alone, *NgAgo* mRNA 0.34 ng/embryo; for NgAgo/ssDNA system, *NgAgo* mRNA 0.34 ng/embryo, gDNA 0.1 nm/embryo; ssDNA alone, gDNA 0.1 nm/embryo; for rescue, *NgAgo* mRNA 0.34 ng/embryo, gDNA 0.1 nm/embryo,

fabp11a mRNA 0.25 ng/embryo.

NaAgo/gDNA and Cas9/gRNA mutation screens by Sanger sequencing

Genomic DNA of zebrafish embryos was extracted at 30 hpf using a genome DNA extraction kit (Ultramicro genotype identification kit from Nanjing YSY Biotech Company) following the manufacturer's protocol. In brief, embryos were resuspended in cell lysis solution and incubated at 65 °C for 30 min, 95 °C for 5 min and 16°C for 1 min. The genomic region surrounding the NgAgo and CRISPR target site for each gene was PCR amplified and some of the PCR products were subcloned into the pGEM®-T Easy vector. The PCR products and pGEM®-T Easy vectors were sent for Sanger sequencing (Shanghai HuaGene Biotech Co., Ltd and Genscript Nanjing). The primers used are listed in Supplementary information, Table S1B.

Statistics

Statistical analysis was performed using GraphPad Prism® software. One-Way ANOVA, T-test, Fisher's exact test, and χ^2 test were used. ($P<0.05$). When we did the One-Way ANOVA for multiple comparisons test, we chose correction for multiple comparisons. The number of embryos used for statistical analysis in Figure 1D: control (n=120); *NgAgo-2nls* mRNA only (n=73); FW-guide 2 only (n=103); FW-guide 2 and *NgAgo-2nls* mRNA (n=274); FW-guide 2 mismatch 1 and *NgAgo-2nls* mRNA (n=90); RV-guide 2 and *NgAgo-2nls* mRNA (n=82); FW-guide 2, RV-guide 2 and *NgAgo-2nls* mRNA (n=153); FW-guide 2, *NgAgo-2nls* mRNA and

fabp11a mRNA injected embryos (n=75). The number of embryos used for statistical analysis in NgAgo mutation experiments, *fabp11a* gDNA+NgAgo-D663A (n=178), NgAgo-D738A (n=88) and NgAgo-D663A-D738A (n=138); *ta* gDNA+NgAgo-D663A (n=160), NgAgo-D738A (n=133) and NgAgo-D663A-D738A (n=121).

Reference

1. **Wang X, Ling CC, Li L, Qin Y, Qi J, Liu X, *et al.* MicroRNA-10a/10b represses a novel target gene mib1 to regulate angiogenesis. *Cardiovascular research* 2016, 110(1): 140-150.**

2. **Huang Y, Wang X, Wang X, Xu M, Liu M, Liu D. Nonmuscle myosin II-B (myh10) expression analysis during zebrafish embryonic development. *Gene expression patterns : GEP* 2013, 13(7): 265-270.**

3. **Jiang Q, Lagos-Quintana M, Liu D, Shi Y, Helker C, Herzog W, *et al.* miR-30a regulates endothelial tip cell formation and arteriolar branching. *Hypertension* 2013, 62(3): 592-598.**

4. **Swarts DC, Hegge JW, Hinojo I, Shiimori M, Ellis MA, Dumrongkulraksa J, *et al.* Argonaute of the archaeon Pyrococcus furiosus is a DNA-guided nuclease that targets cognate DNA. *Nucleic Acids Res* 2015, 43(10): 5120-5129.**

> NgAgo-2nls sequence information

(Start codon)

GGATCCGCCACCATGGTGCCCAAGAAGAAGAGAAAGGTGGCTACCGTGAT

CGACCTGGATAGCACAACAACAGCTGACGAGCTGACATCAGGACATACAT

ACGACATCAGCGTGACACTGACAGGAGTTTACGACAACACAGACGAGCAG

CATCCTAGAATGTCTCTGGCTTTCGAGCAGGATAACGGAGAAAGACGCTAC

ATCACCCTCTGGAAGAACACAACCCCTAAGGACGTGTTCACATACGACTAC

GCTACAGGAAGCACCTACATCTTCACCAACATCGACTACGAGGTGAAGGA

CGGATACGAGAACCTGACAGCTACATACCAGACCACAGTGGAAAACGCTA

CAGCTCAGGAAGTGGGAACAACAGACGAAGACGAGACATTTGCAGGAGG

AGAACCACTGGATCATCATCTGGACGACGCTCTGAACGAAACACCAGACG

ACGCAGAAACAGAGTCAGATAGCGGACACGTGATGACATCTTTCGCTTCTA

GAGATCAGCTCCCAGAGTGGACACTGCACACATACACACTGACAGCTACC

GACGGAGCTAAAACAGACACAGAGTACGCTAGGAGAACACTGGCTTACAC

AGTGAGACAGGAGCTGTATACAGATCACGACGCAGCTCCAGTTGCTACAG

ACGGACTGATGCTGCTGACACCAGAACCTCTGGGAGAAACACCTCTGGAT

CTGGATTGCGGAGTTAGAGTGGAAGCAGACGAGACAAGAACACTGGACTA

CACCACAGCTAAGGATAGACTGCTGGCAAGAGAGCTGGTTGAAGAGGGAC

TGAAAAGAAGCCTCTGGGACGATTACCTGGTTAGAGGAATCGACGAGGTG

CTGTCTAAAGAGCCAGTTCTGACTTGCGACGAGTTTGATCTGCACGAGAGA

TACGACCTGAGCGTTGAAGTTGGACATAGCGGAAGAGCTTACCTGCACATC

AACTTCAGGCACAGGTTCGTGCCTAAACTGACACTGGCAGATATCGACGAC

GATAACATCTACCCAGGACTGAGAGTGAAGACCACATACAGACCTAGGAG

AGGACACATTGTTTGGGGACTGAGAGACGAGTGCGCTACAGATAGCCTGA

ACACACTGGGAAACCAGTCAGTGGTGGCTTACCACAGAAACAACCAGAC

ACCTATCAACACAGATCTGCTGGACGCTATCGAAGCAGCAGATAGAAGAGT

GGTGGAGACAAGAAGACAGGGACACGGAGACGACGCAGTTAGCTTTCCT

CAGGAACTGCTGGCAGTTGAACCTAACACCCACCAGATCAAGCAGTTCGC

TTCAGACGGATTTCATCAGCAGGCTAGAAGCAAAACAAGACTGTCCGCTA

GCCGCTGTTCAGAGAAAGCTCAGGCTTTTGCCGAGAGACTGGACCCTGTT

AGACTGAACGGAAGCACAGTTGAGTTTAGCAGCGAGTTCTTCACCGGAAA

CAACGAACAGCAGCTGAGACTCCTGTACGAGAACGGAGAAAGCGTGCTG

ACATTCAGAGACGGAGCTAGAGGAGCTCATCCAGACGAAACATTCAGCAA

GGGAATCGTGAACCCTCCAGAGTCTTTTGAAGTGGCAGTGGTTCTGCCAG

AACAGCAGGCAGATACTTGCAAAGCTCAGTGGGACACAATGGCAGATCTG

CTGAATCAGGCAGGAGCTCCTCCTACAAGAAGCGAAACAGTGCAGTACGA

CGCTTTTAGCTCTCCAGAAAGCATCTCTCTGAACGTGGCAGGAGCTATTGA

TCCTTCAGAGGTGGACGCAGCATTTGTTGTTCTGCCTCCAGATCAGGAGGG

ATTTGCAGATCTGGCTTCTCCTACAGAGACATACGACGAACTGAAGAAGGC

TCTGGCTAACATGGGAATCTACAGCCAGATGGCTTACTTCGACAGGTTCAG

AGACGCCAAGATCTTCTACACCAGGAATGTTGCTCTGGGACTGCTGGCAGC

AGCAGGAGGAGTTGCTTTTACAACAGAGCACGCTATGCCAGGAGACGCAG

ATATGTTCATCGGAATCGACGTGAGCAGATCTTATCCAGAGGACGGAGCTT

CAGGACAGATTAACATCGCAGCTACAGCTACAGCCGTGTATAAAGACGGAA

CCATCCTGGGACACTCTTCTACAAGACCTCAGCTGGGAGAGAAACTGCAG

TCTACAGACGTGAGGGACATCATGAAGAACGCTATCCTGGGATACCAGCAG

GTTACAGGAGAGTCTCCTACACACATCGTGATCCACAGAGACGGATTCATG

AACGAGGACCTGGACCCTGCTACAGAGTTTCTGAACGAGCAGGGAGTGGA

GTACGATATCGTGGAGATCAGAAAGCAGCCTCAGACAAGACTGCTGGCAG

TTTCAGACGTGCAGTACGATACACCAGTGAAGAGCATCGCAGCTATCAACC

AGAACGAGCCTAGAGCTACAGTTGCTACATTCGGAGCTCCAGAGTATCTGG

CTACAAGAGATGGAGGAGGACTGCCTAGACCTATTCAGATCGAGAGAGTG

GCAGGAGAAACAGACATCGAGACACTGACAAGACAGGTGTATCTGCTGTC

TCAGTCTCACATCCAGGTGCATAACAGCACAGCTAGACTGCCTATCACCAC

AGCTTACGCAGATCAGGCTAGCACACACGCTACAAAAGGATACCTGGTGC

AGACAGGAGCTTTTGAGTCTAACGTGGGATTCCTGAAGAGACCAGCAGCT

ACAAAGAAGGCAGGACAGGCTAAGAAGAAGAAGTGACTCGAG

(Stop codon)
