# Supplementary material for: NgAgo-based fabp11a gene knockdown causes eye developmental defects in zebrafish
Source: Cell Res. 2016 Nov 11;26(12):1349–52. doi: 10.1038/cr.2016.134 (PMC5143420; doi:10.1038/cr.2016.134)
Supplement: Supplementary information, Table S1 — The sequences were used for fabp11a [file cr2016134x2.pdf]

Supplementary information, Table S1A

**The sequences were used for *fabp11a***	
Morpholino	5’-ACCATTTTCCCGAATAATGATGCTC-3’
Control morpholino	5’-CCTCTTACCTCAGTTACAATTTATA-3’
gRNA 1	5’-GTGCAGAAACAGACCTGGGA-3’
gRNA 2	5’-GGAGTCCACAATAGAGAGAG-3’
RV-guide DNA 1	5’-AGCCGTTCGAGGAGACCACGGCGG-3’
FW-guide DNA 1	5‘-CCGTGGTCTCCTCGAACGGCTCGT-3’
RV-guide DNA 2	5’-TGTGCAGAAACAGACCTGGGACGG-3’
FW-guide DNA 2	5’-TCCCAGGTCTGTTTCTGCACAAGT-3’
FW-guide DNA 2-mismatch1	5’-TCAAAGGTCTGTTAATTCACAAGT-3’
FW-guide DNA 2-mismatch2	5’-TCTTAGGTCTGTAAATGCACAAGT-3’
FW-guide DNA 2-20bp	5’-AGGTCTGTTTCTGCACAAGT-3’
FW-guide DNA 2-21bp	5’-CAGGTCTGTTTCTGCACAAGT-3’
FW-guide DNA 2-22bp	5’-CCAGGTCTGTTTCTGCACAAGT-3’
FW-guide DNA 2-23bp	5’-CCCAGGTCTGTTTCTGCACAAGT-3’
FW-guide DNA 2-25bp	5’-GTCCCAGGTCTGTTTCTGCACAAGT-3’
FW-guide DNA 2-26bp	5’-CGTCCCAGGTCTGTTTCTGCACAAGT-3’
FW-guide DNA 2-27bp	5’-CCGTCCCAGGTCTGTTTCTGCACAAGT-3’
FW-guide DNA 3	5’-GTGTGGGTTTTGCTACTCGTCAGG-3’
FW-guide DNA 4	5’-CAAACCCAATCTAGTGGTGTGTGT-3’

**The sequences were used for *ntl(ta)***	
RV-guide DNA 1	5’-CTCGACCCTAATGCAATGTACTCG-3’
FW-guide DNA 1	5’-CGAGTACATTGCATTAGGGTCGAG-3’
FW-guide DNA 1 mismatch1	5’-CGAGTACATAACATTAATTTCGAG-3’
FW-guide DNA 1 mismatch2	5’-CGAGTATGTTGCATTATAATCGAG-3’
FW-guide DNA 1-20bp	5’-TACATTGCATTAGGGTCGAG-3’
FW-guide DNA 1-21bp	5’-GTACATTGCATTAGGGTCGAG-3’
FW-guide DNA 1-22bp	5’-AGTACATTGCATTAGGGTCGAG-3’
FW-guide DNA 1-23bp	5’-GAGTACATTGCATTAGGGTCGAG-3’
FW-guide DNA 1-25bp	5’-CCGAGTACATTGCATTAGGGTCGAG-3’
FW-guide DNA 1-26bp	5’-ACCGAGTACATTGCATTAGGGTCGAG-3’
FW-guide DNA 1-27bp	5’-GACCGAGTACATTGCATTAGGGTCGAG-3’
**The sequences were used for *kdrl***	
FW-guide DNA 1	5 ‘-ACCAAAGCAGACCCCTCAAACCAG-3’
**The sequences were used for *flt1***	
FW-guide DNA 1	5 ‘- CACACGGGCTCCTACCGCTGCAGG-3’
Morpholino	5 ‘- ATATCGAACATTCTCTTGGTCTTGC-3’
Control morpholino	5’-CCTCTTACCTCAGTTACAATTTATA-3’
**The sequences were used for *lama1***	
FW-guide DNA 1	5 ‘- CATCATCATTAAAGCAGCAAACTC-3’

Supplementary information, Table S1B

**Primers for *fabp11a***		
	Left primer	Right primer
RT-PCR and probe	5'-TGCTACTCGTCAGGTTGGAA-3'	5'-ACTGAGCCTGGCATCTTCAA-3'
Real Time PCR	5'-ACGGCGGATGACAGAAAGA-3'	5'-CACCACATCACCCATCTTGC-3'
Mutation Screen	5'-TGACCACCAGCGACAAC-3'	5'-ATACAGCCTGCTATTTAA-3'
Sequencing primer	5'-AACTATGGGTATGTGGAC-3'	
**Primers for *ta (ntl)***		
Real Time	5'-ATCCCAGCCATTACTCCCAC-3'	5'-GGTAGTGTTTGTGGTGTGGG-3'
Mutation Screen	5'-TCACCAAGACTGGGAGG-3'	5'-CCACCGACTTTCACGAT-3'
Sequencing primer1	5'-GCCGAAGTTGGGTGAGTCCG-3'	
Sequencing primer2	5'-CTGTCCTCCTCCGTTGAGTT-3'	
**Primers for *kdrl***		
Real Time PCR	5'-ATCCCAGCCATTACTCCCAC-3'	5'-TGCCTCAAGTTGTGTATGACT -3'
Mutation Screen	5'-CAACACAACATCCAAACAAAACA -3'	5'-AGGGGCCCTTAATGCTACG-3'

Sequencing1	5 ‘- TCCATAATAACACCATATCC-3’	
Sequencing2	5 ‘- GCTTGAATGAATAATGATCG-3’	
**Primers for *flt1***		
Real Time PCR	5’-TACATGAAGGAGGGAGAGCC-3’	5’-ATGAATCCCTGCCTGCTGTT -3’
Mutation Screen	5’-ACCCTCAAGCAAGACCAAGA -3’	5’-CTGATGGACACCCTGGAGTT-3’
Sequencing1	5 ‘- GGGACGGTGGGAGCTCCAGT -3’	
Sequencing2	5 ‘- AACTCTACCCCGACACACAT -3’	
**Primers for *lama1***		
Real Time PCR	5’- AGTACTGCTGCCTGATGACC -3’	5’-GAACTGGTGGGATTCTGGGA-3’
Mutation Screen	5’- TGCAAATAGTTCTGACTTGA-3’	5’-ATCGCTAATGGCGTAAA-3’
Sequencing	5 ‘- CTGACTTGAACTGTAAATACAT -3’	
