# Supplementary material for: NgAgo-based fabp11a gene knockdown causes eye developmental defects in zebrafish
Source: Cell Res. 2016 Nov 11;26(12):1349–52. doi: 10.1038/cr.2016.134 (PMC5143420; doi:10.1038/cr.2016.134)
Supplement: Supplementary information, Figure S1 — NgAgo based fabp11a gene knockdown caused eye developmental defects in zebrafish. [file cr2016134x3.pdf]

Supplementary information, Figure S1

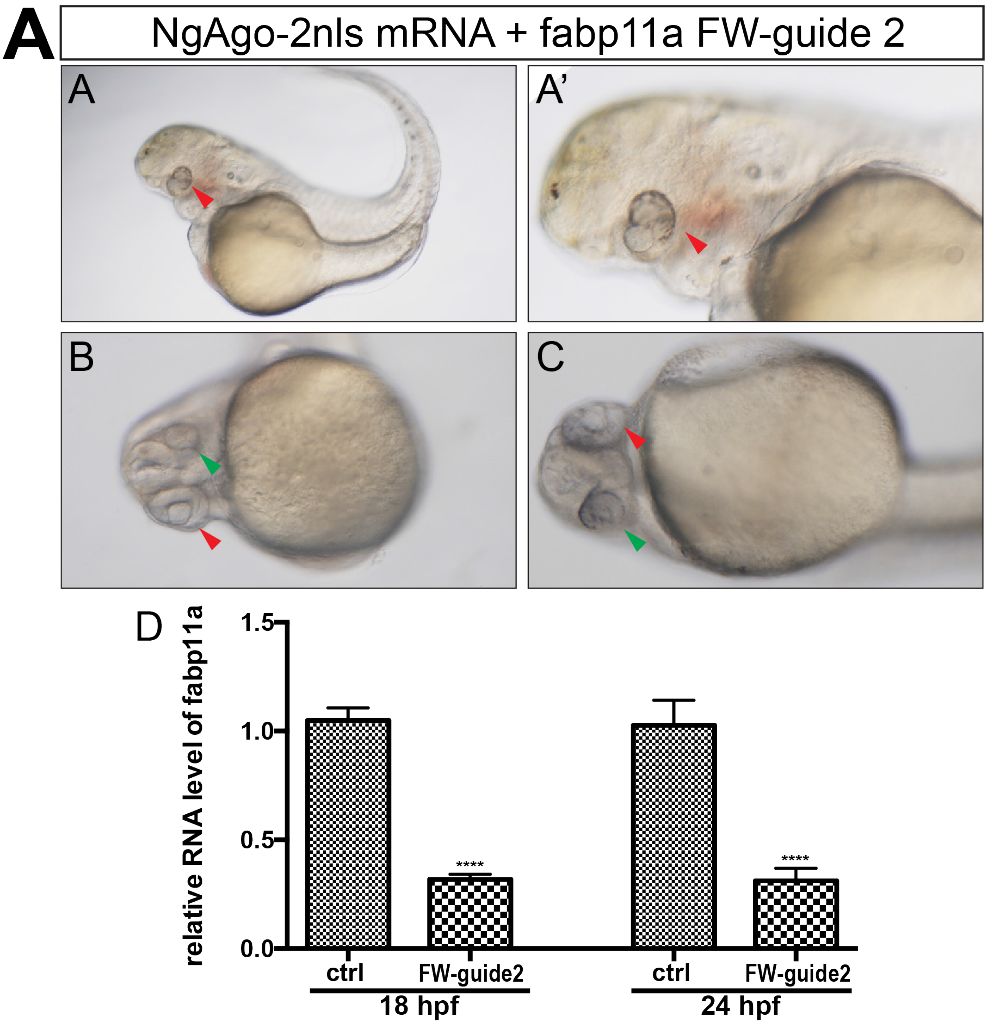

Figure S1A. NgAgo based *fabp11a* gene knockdown caused eye developmental defects in zebrafish. (A, A') Microscopy analysis of eyes in *fabp11a* FW-guide DNA 2 and *NgAgo-2nls* mRNA coinjected embryos, lateral view. Red arrowheads indicate small developmental abnormal eyes. (B, C) Microscopy analysis of eyes in *fabp11a* FW-guide DNA 2 and *NgAgo-2nls* mRNA coinjected embryos, ventral view. Red arrowhead indicates relative normal eye. Green arrowhead indicates small developmental abnormal eye. (D) Relative mRNA levels of zebrafish *fabp11a* in 18 and 24 hpf control and *ta* FW-guide DNA 2 and *NgAgo-2nls* mRNA coinjected embryos. T-test; ****, $P<0.0001$.

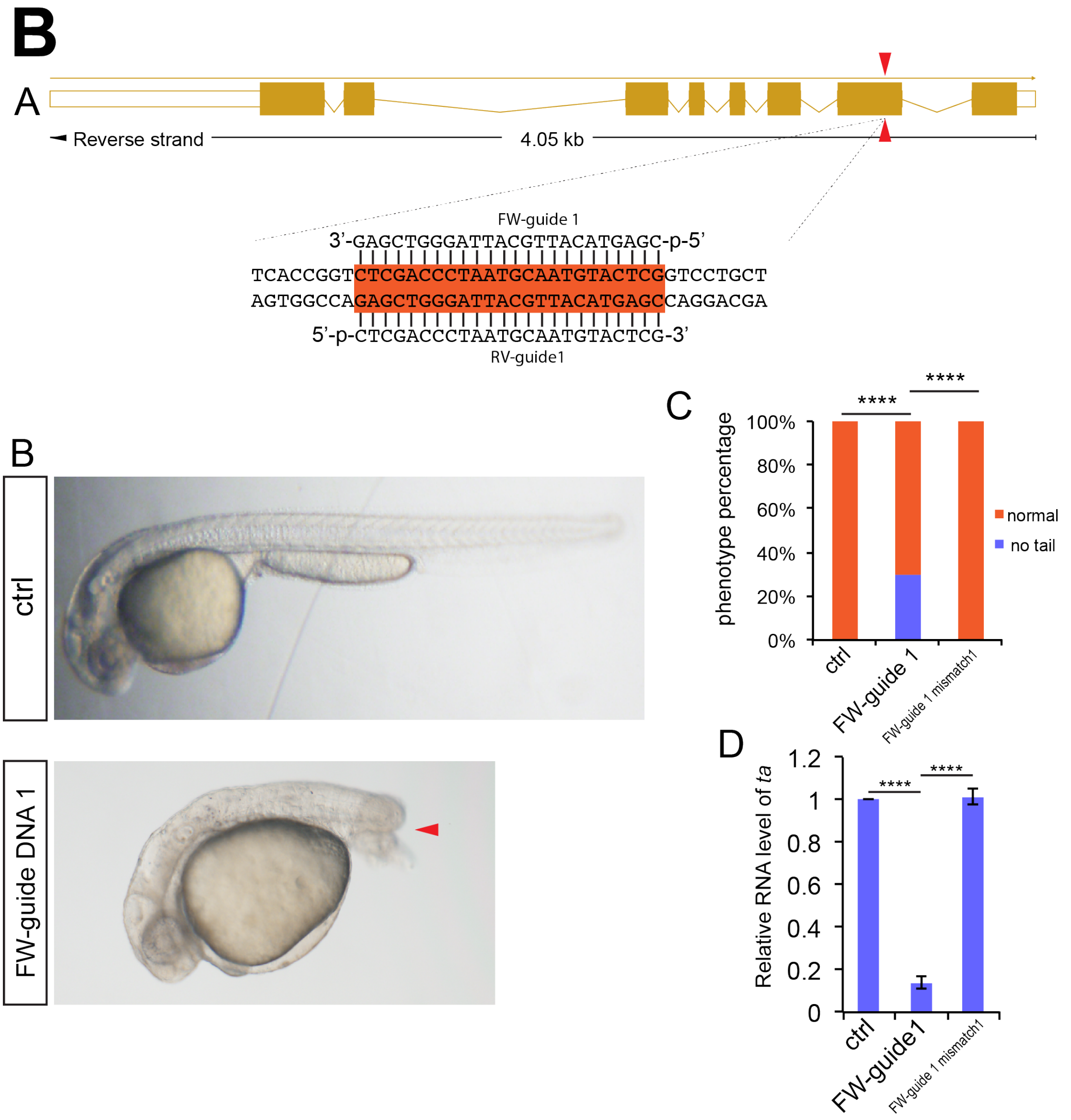

Figure S1B. NgAgo based *ta* gene knockdown caused no tail phenotype in zebrafish. (A) Schematic diagram showing Guide DNA targeting sites on the exon of *ta* gene. Guide DNA targeting sites are indicated by arrowheads. The targeting sequences are highlighted in orange. (B) Microscopy analysis of tail in control, *NgAgo-2nls* and *ta* FW-guide DNA 1 coinjected, and *NgAgo-2nls* and *ta* mismatch guide DNA coinjected embryos, lateral view. Red arrowhead indicates disrupted tail. (C) Statistical analysis of phenotype efficiency in control, *NgAgo-2nls* and *ta* FW-guide DNA 1 coinjected, and *NgAgo-2nls* and *ta* mismatch guide DNA coinjected embryos. χ^2 test; ****, *P*<0.0001. (D) Relative mRNA levels of zebrafish *ta* in 30 hpf control and *ta* FW-guide DNA 1 and *NgAgo-2nls* mRNA coinjected embryos. T-test; ****, *P*<0.0001.

C

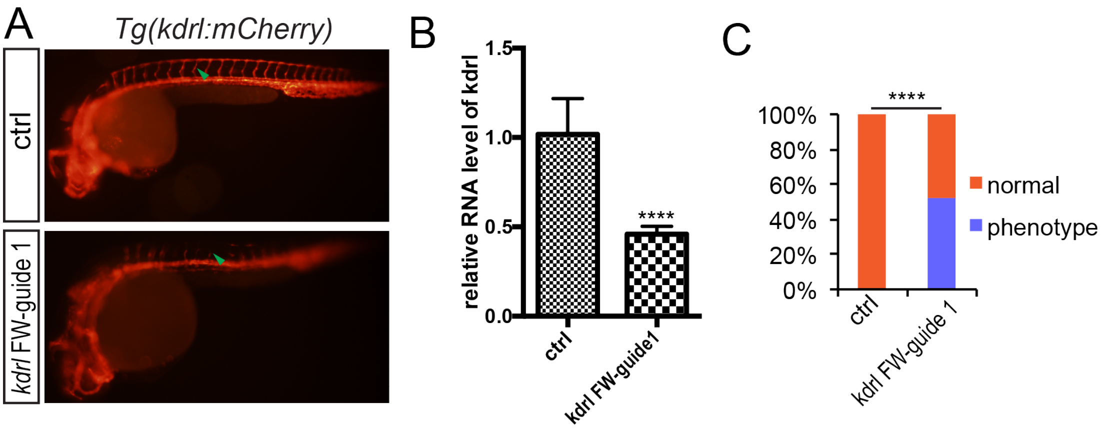

Figure S1C. NgAgo based *kdrl* gene knockdown caused angiogenic defects phenotype in zebrafish. (A) Microscopy analysis of blood vessel in control and *NgAgo-2nls* and *kdrl* FW-guide DNA 1 coinjected embryos, lateral view. Green arrowhead indicates intersegmental vessel (ISVs). (B) Relative mRNA levels of zebrafish *kdrl* in 30 hpf control and *kdrl* FW-guide DNA 1 and *NgAgo-2nls* mRNA coinjected embryos. T-test; ****, P<0.0001. (C) Statistical analysis of phenotype efficiency in control and *NgAgo-2nls* and *kdrl* FW-guide DNA 1 coinjected embryos. χ^2 test; ****, P<0.0001. The *Tg(kdrl:mCherry)* transgenic line was described in our previous work (Jiang Q, Lagos-Quintana M, Liu D, Shi Y, Helker C, Herzog W, et al. miR-30a regulates endothelial tip cell formation and arteriolar branching. Hypertension 2013, 62(3): 592-598.).

D

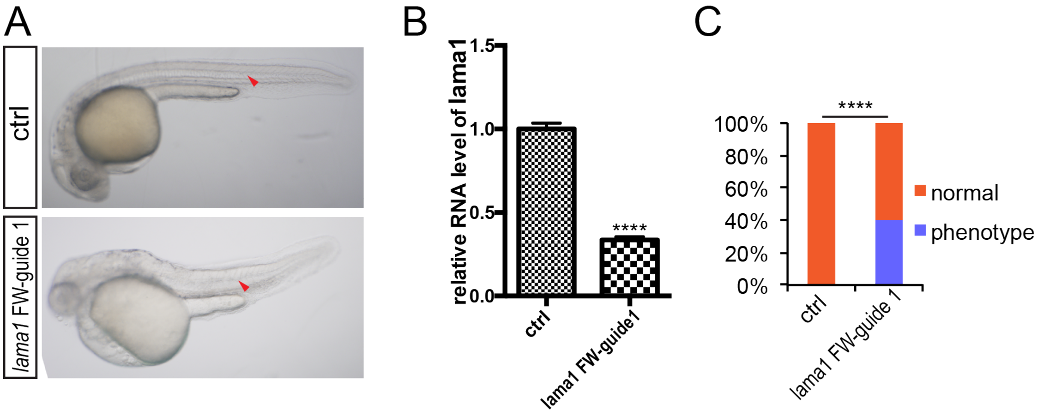

Figure S1D. NgAgo based *lama1* gene knockdown caused notochord defects phenotype in zebrafish. (A) Microscopy analysis of notochord in control and *NgAgo-2nls* and *lama1* FW-guide DNA 1 coinjected embryos, lateral view. Red arrowhead indicates notochord. (B) Relative mRNA levels of zebrafish *lama1* in 30 hpf control and *lama1* FW-guide DNA 1 and *NgAgo-2nls* mRNA coinjected embryos. T-test; ****, $P<0.0001$. (C) Statistical analysis of phenotype efficiency in control and *NgAgo-2nls* and *lama1* FW-guide DNA 1 coinjected embryos. χ^2 test; ****, $P<0.0001$.

E

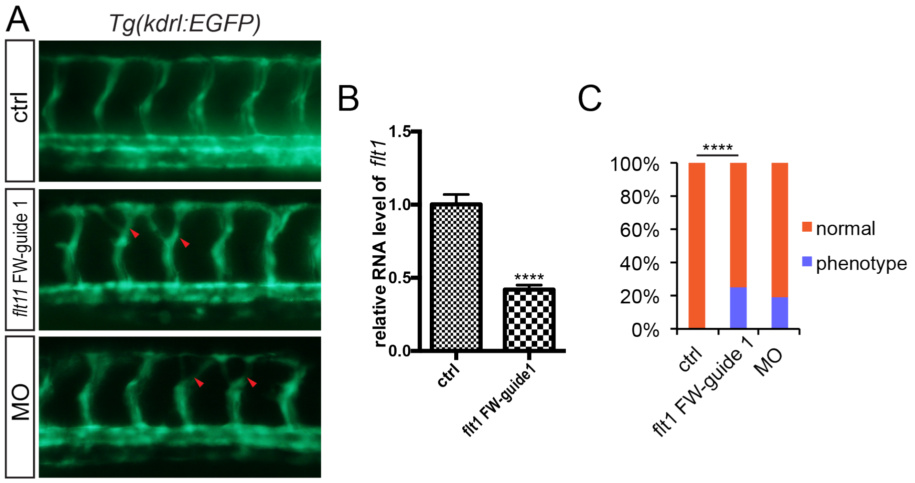

Figure S1E. NgAgo based *flt1* gene knockdown caused excessive angiogenesis phenotype in zebrafish. (A) Microscopy analysis of blood vessel in control and *NgAgo-2nls* and *flt1* FW-guide DNA 1 coinjected embryos, lateral view. Green arrowhead indicates intersegmental vessel (ISVs). (B) Relative mRNA levels of zebrafish *flt1* in 30 hpf control and *flt1* FW-guide DNA 1 and *NgAgo-2nls* mRNA coinjected embryos. T-test; ****, P<0.0001. (C) Statistical analysis of phenotype efficiency in control and *NgAgo-2nls* and *flt1* FW-guide DNA 1 coinjected embryos. χ^2 test; ****, P<0.0001. The *Tg(kdrl:EGFP)* transgenic line was described in our previous work (Wang X, Ling CC, Li L, Qin Y, Qi J, Liu X, et al. MicroRNA-10a/10b represses a novel target gene mib1 to regulate angiogenesis. Cardiovascular research 2016, 110(1): 140-150.).

F

A

fabp11a FW-guide2

5′-AGGTCTGTTTCTGCACAAGT-3′	20 bp
5′-CAGGTCTGTTTCTGCACAAGT-3′	21 bp
5′-CCAGGTCTGTTTCTGCACAAGT-3′	22 bp
5′-CCCAGGTCTGTTTCTGCACAAGT-3′	23 bp
5′-TCCCAGGTCTGTTTCTGCACAAGT-3′	24 bp
5′-GTCCCAGGTCTGTTTCTGCACAAGT-3′	25 bp
5′-CGTCCCAGGTCTGTTTCTGCACAAGT-3′	26 bp
5′-CCGTCCCAGGTCTGTTTCTGCACAAGT-3′	27 bp

FW-guide2	20 bp	21 bp	22 bp	23 bp	24 bp	25 bp	26 bp	27 bp
Phenotype	54%	49%	21%	18%	28%	9%	12%	11%

B

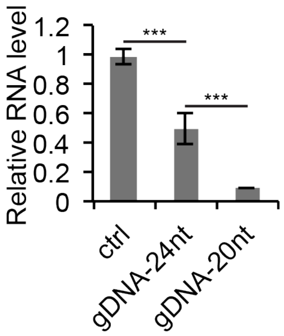

C

ta FW-guide1

5′-TACATTGCATTAGGGTCGAG-3′	20 bp
5′-GTACATTGCATTAGGGTCGAG-3′	21 bp
5′-AGTACATTGCATTAGGGTCGAG-3′	22 bp
5′-GAGTACATTGCATTAGGGTCGAG-3′	23 bp
5′-CGAGTACATTGCATTAGGGTCGAG-3′	24 bp
5′-CCGAGTACATTGCATTAGGGTCGAG-3′	25 bp
5′-ACCGAGTACATTGCATTAGGGTCGAG-3′	26 bp
5′-GACCGAGTACATTGCATTAGGGTCGAG-3′	27 bp

FW-guide1	20 bp	21 bp	22 bp	23 bp	24 bp	25 bp	26 bp	27 bp
Phenotype	47%	45%	39%	29%	33%	21%	18%	16%

Figure S1F. Phenotype efficiency in NgAgo-2nls and different length of guide DNAs coinjected embryos. (A) Phenotype efficiency in NgAgo-2nls and different length of *fabp11a* guide DNAs coinjected embryos. (B) Relative mRNA levels of zebrafish *fabp11a* in control, 24nt gDNA and *NgAgo-2nls* mRNA coinjected embryos, 20nt gDNA and *NgAgo-2nls* mRNA coinjected embryos. One-Way ANOVA; ***, $P<0.001$. (C) Phenotype efficiency in NgAgo-2nls and different length of *ta* guide DNAs coinjected embryos.

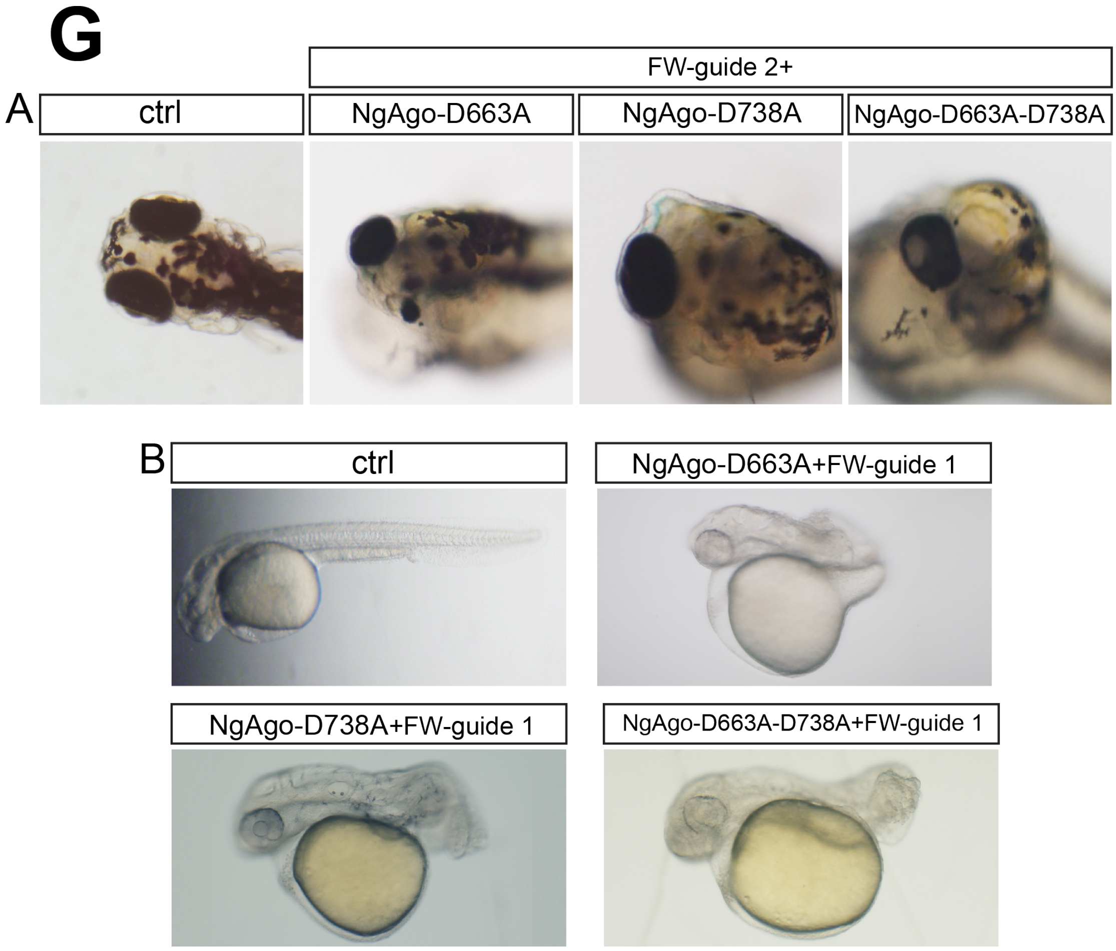

Figure S1G. Mutated NgAgo mRNA coinjected with *fabp11a* FW-gDNA2 and *ta* FW-gDNA1 respectively caused eye phenotype and no tail phenotype. (A) Microscopy analysis of eyes in ctrl, *fabp11a* FW-guide DNA 2 and NgAgo-D663A mRNA coinjected, *fabp11a* FW-guide DNA 2 and NgAgo-D738A mRNA coinjected, *and fabp11a* FW-guide DNA 2 and NgAgo-D663A-D738A mRNA coinjected embryos. (B) Microscopy analysis of eyes in ctrl, *ta* FW-guide DNA 1 and NgAgo-D663A mRNA coinjected, *ta* FW-guide DNA 1 and NgAgo-D738A mRNA coinjected, *and ta* FW-guide DNA 1 and NgAgo-D663A-D738A mRNA coinjected embryos.

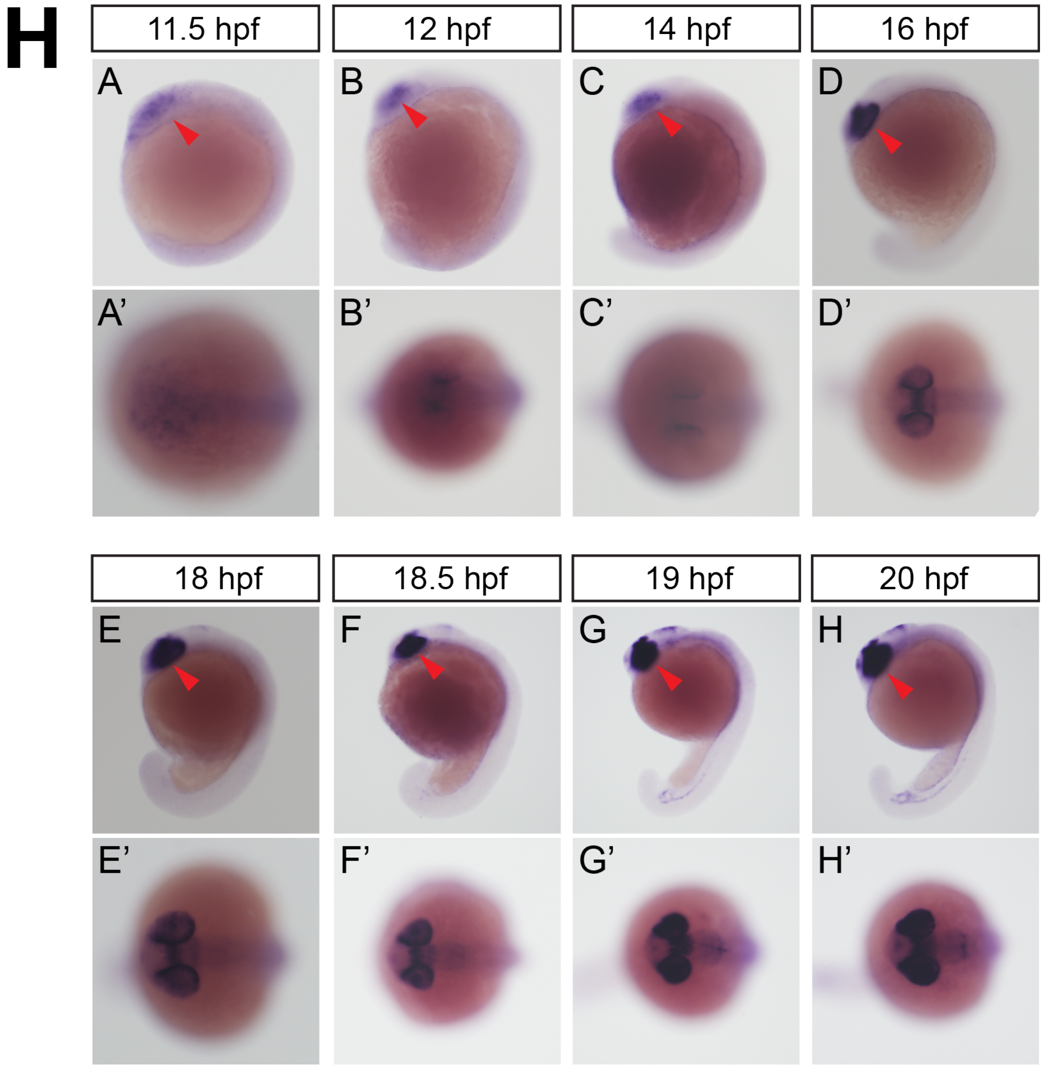

Figure S1H. Whole mount *in situ* hybridization analysis of zebrafish embryos using antisense *fabp11a* probe. (A) 11.5 hpf, lateral view. Red arrowhead indicates eye field. (A'). 11.5 hpf, coronal view. (B) 12 hpf, lateral view. Red arrowhead indicates eye field. (B'). 12 hpf, coronal view. (C) 14 hpf, lateral view. Red arrowhead indicates eye field. (C'). 14 hpf, coronal view. (D) 16 hpf, lateral view. Red arrowhead indicates eye field. (D'). 16 hpf, coronal view. (E) 18 hpf, lateral view. Red arrowhead indicates eye field. (E'). 18 hpf, coronal view. (F) 18.5 hpf, lateral view. Red arrowhead indicates eye field. (F'). 18.5 hpf, coronal view. (G) 19 hpf, lateral view. Red arrowhead indicates eye field. (G'). 19 hpf, coronal view. (H) 20 hpf, lateral view. Red arrowhead indicates eye field. (H'). 20 hpf, coronal view.

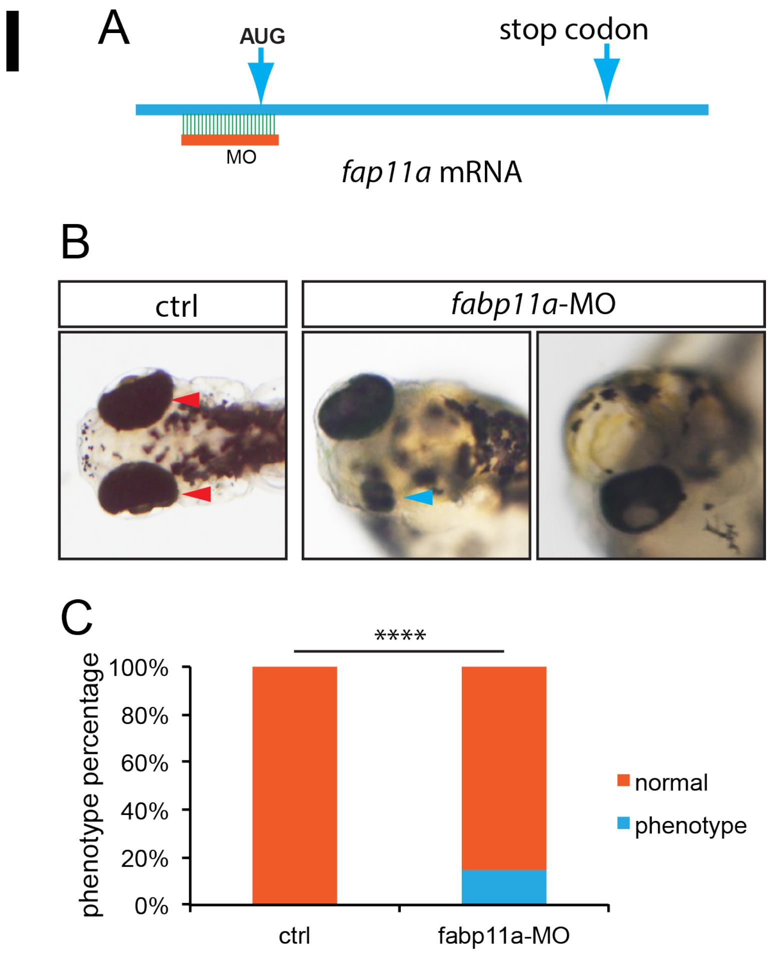

Figure S1I. *Fabp11a* gene knockdown by morpholino resulted in eye developmental defects in zebrafish. (A) Diagram of *fabp11a* translation blocking morpholino design. (B) Microscopy analysis of eyes in control and *fabp11a* morphants, dorsal view. Red arrowheads indicate normal paired eyes. Blue arrowhead indicates normal small eyes. (C) Statistical analysis of phenotype efficiency in control and *fabp11a* MO injected embryos. χ^2 test; ****, $P<0.0001$.

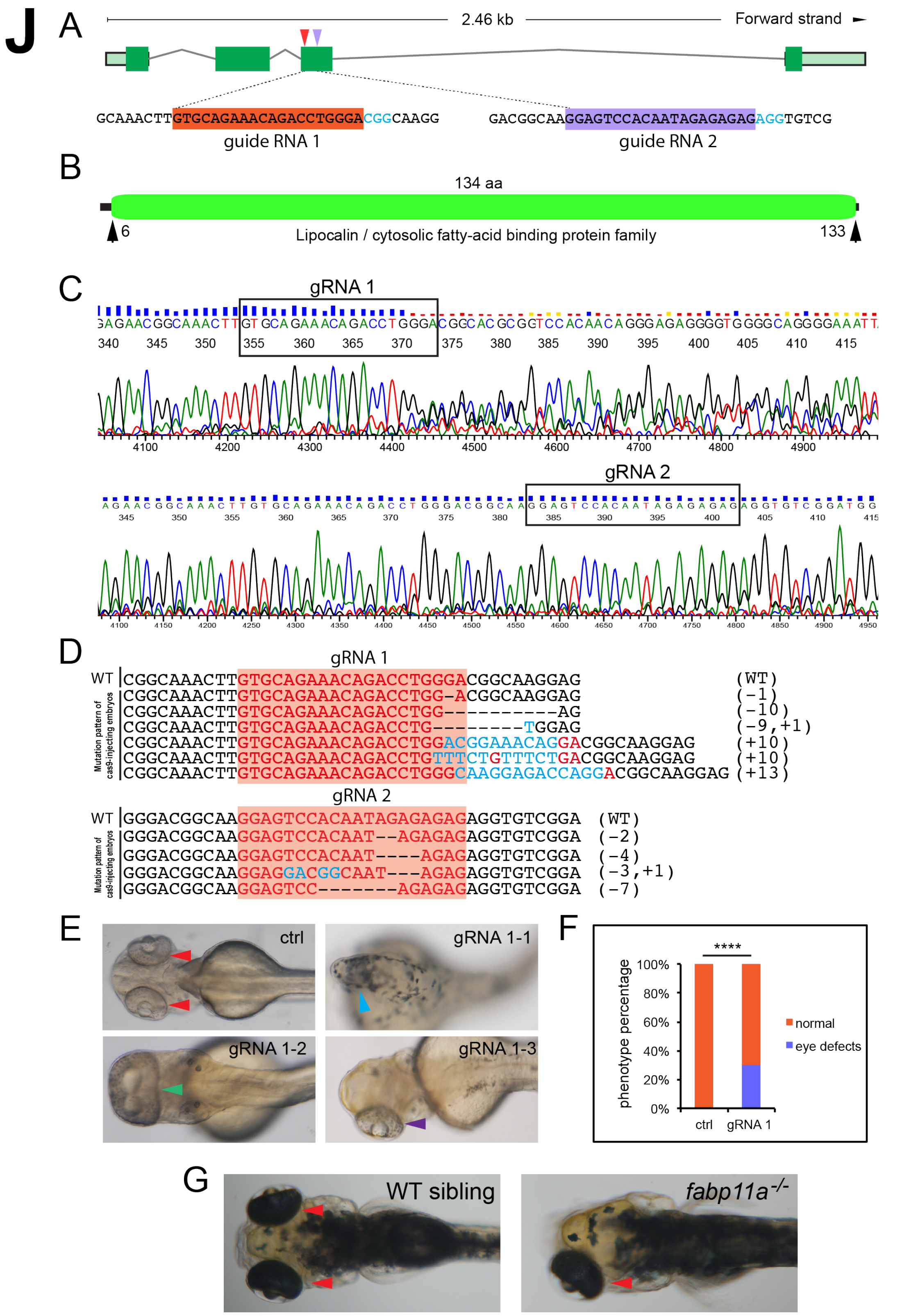

Figure S1J. *Fabp11a* gene knockout using CRISPR/Cas9 system caused eye developmental defects in zebrafish. (A) Schematic diagram showing Guide RNA targeting sites on the exons of *fabp11a* gene. Guide DNA targeting sites are indicated by arrowheads. The targeting sequences are highlighted in orange and purple. (B)

Graphical view of Fabp11a protein domain. (C) Sanger sequencing analysis of PCR fragments amplified from gRNA1 and gRNA2 target regions in gRNA1/gRNA2 and *cas9* mRNA coinjected embryos. (D) Mutation pattern of gRNA1/gRNA2 and *cas9* mRNA coinjected embryos. Numbers in the brackets show the number of nucleotides were deleted (-) or inserted (+). Inserted nucleotide is in red. WT, wild type. (E) Microscopy analysis of eyes in control and gRNA1-*cas9* mRNA coinjected embryos, dorsal view. Red arrowheads indicate normal paired eyes. Blue arrowhead indicates no eye in gRNA1-*cas9* mRNA coinjected embryo. Green arrowhead indicates single fused big eyes, dorsal view. Purple arrowhead indicates single eye, dorsal view. (F) Statistical analysis of phenotype efficiency in control and gRNA1-*cas9* mRNA coinjected embryos. χ^2 test; ****, $P<0.0001$. (G) Microscopy analysis of eyes in control and *fabp11a*$^{-/-}$ embryos. Arrowheads indicate eyes.
